# Acceptability of receiving HIV and sexual and reproductive health services at hair salons among university students in Zambia: a cross-sectional survey

**DOI:** 10.64898/2026.08.06.26359879

**Published:** 2026-08-10

**Authors:** Malena Chiaborelli, Lilian Nayame, Twaambo E. Hamoonga, Oliver Mweemba, Mamaswatsi Kopeka, Karen Hampanda, Alain Amstutz

## Abstract

Adolescent girls and young women in Zambia face barriers to accessing HIV and sexual and reproductive health services. We conducted a cross-sectional survey among 846 female students at a university in Zambia to assess acceptability of receiving these services in hair salons and explore whether HIV acquisition risk influenced acceptability of HIV services. Acceptability varied by service, ranging from 25% to 50%. Higher HIV acquisition risk may increase the acceptability for HIV services. Hair salons may be a promising community-based, demedicalized setting for delivering selected HIV and sexual and reproductive health services to HIV at-risk female students.

## Full Text Availability

The license terms selected by the author(s) for this preprint version do not permit archiving in PMC. The full text is available from the preprint server.

